# Departures From Optimality When Pursuing Multiple Approach or Avoidance Goals

**DOI:** 10.1037/apl0000082

**Published:** 2016-03-10

**Authors:** Timothy Ballard, Gillian Yeo, Andrew Neal, Simon Farrell

**Affiliations:** 1 Business School, University of Western Australia and School of Experimental Psychology, University of Bristol; 2 Business School, University of Western Australia; 3 School of Psychology, University of Queensland; 4 School of Psychology, University of Western Australia and School of Experimental Psychology, University of Bristol

**Keywords:** approach-avoidance, multiple-goals, decision making, risk, prioritization

## Abstract

This article examines how people depart from optimality during multiple-goal pursuit. The authors operationalized optimality using dynamic programming, which is a mathematical model used to calculate expected value in multistage decisions. Drawing on prospect theory, they predicted that people are risk-averse when pursuing approach goals and are therefore more likely to prioritize the goal in the best position than the dynamic programming model suggests is optimal. The authors predicted that people are risk-seeking when pursuing avoidance goals and are therefore more likely to prioritize the goal in the worst position than is optimal. These predictions were supported by results from an experimental paradigm in which participants made a series of prioritization decisions while pursuing either 2 approach or 2 avoidance goals. This research demonstrates the usefulness of using decision-making theories and normative models to understand multiple-goal pursuit.

Pursuing multiple, competing goals is a pervasive feature of modern life. We all have to manage competing demands for our time and are frequently faced with situations where we have to make trade-offs. Imagine you have two projects to complete but will only have a limited number of opportunities to work on them before they are due. You are uncertain whether you can complete both by the deadline. When given the opportunity to work on one of the projects, do you (a) focus on the one that’s in the best shape in the hope of completing at least one project on time, but risk missing an opportunity to complete both; or (b) focus on the one that’s in the worst shape to have a chance of completing both projects, at the risk of completing neither? The optimal choice provides the most efficient allocation of resources (Keeney & Raiffa, 1976). Yet, identifying the optimal choice is complex because it requires anticipating how actions taken now will affect the potential for future goal attainment.

Studies that have examined multiple-goal pursuit have identified factors that predict how people prioritize competing goals (Kernan & Lord, 1990; Louro, Pieters, & Zeelenberg, 2007; Schmidt & DeShon, 2007; Schmidt, Dolis, & Tolli, 2009; Schmidt & Dolis, 2009). However, this work has not examined whether people make prioritization decisions that are optimal. Comparing observed behavior to an optimal criterion allows one to identify biases, which are systematic deviations from that criterion. Biases provide insight into the psychological processes that underlie decision making (Kahneman & Tversky, 1979). The examination of decision biases has lead to the development of highly sophisticated models of decision making that are capable of accounting for a wide range of empirical phenomena (Busemeyer, 2015). Identifying biases also provides an opportunity to enhance decision making in practice. For example, decision support systems based on models of optimal decision making have been implemented to help investors overcome biases to improve returns (e.g., Bhandari & Hassanein, 2012).

To address this gap, we implement a normative model of decision making during multiple-goal pursuit. Normative models provide a standard for evaluating behavior, rather than predicting the behavior itself (Baron, 2004, 2012). Thus, they are ideal for examining the optimality of prioritization decisions. We define the optimal decision according to expected utility theory (von Neumann & Morgenstern, 1947), as that which maximizes the expected value of an outcome (in this case, the number of goals achieved). In the sections below, we first operationalize multiple-goal pursuit as a multistage decision task. We then show how dynamic programming (Bellman, 1966)—a method for calculating the expected value of actions in multistage decision tasks—can be used to derive a normative model of prioritization.

We then use prospect theory to make predictions about how people depart from optimality when pursuing approach and avoidance goals. An approach goal represents a desired state that the person strives to achieve, whereas an avoidance goal represents an undesired state that the person strives to avoid (Carver & Scheier, 1990, 1998; Elliot & Covington, 2001). According to prospect theory (Kahneman & Tversky, 1979), people make decisions that are risk-averse when given the opportunity to gain, yet risk-seeking when faced with the threat of loss. However, this theory has typically been applied to static, single-stage decisions, and these findings do not always replicate in dynamic environments (Hollenbeck, Ilgen, Phillips, & Hedlund, 1994; Slattery & Ganster, 2002; Thaler & Johnson, 1990). Using the normative model as a benchmark, we examine whether people are risk-averse when pursuing multiple approach goals, and risk-seeking when pursuing multiple avoidance goals.

## Multistage Decision Tasks

We examine departures from optimality using a multistage decision task. A multistage decision is a task that is broken down into a series of discrete stages (Rapoport & Wallsten, 1972). The stages are discrete because the individual can only act on the environment at particular times, then must wait for the action to affect the environment (or not) before deciding on the next course of action. There are many situations in which people have to manage discrete tasks and can only adjust their priorities at particular times. For example, a project manager allocating resources across multiple projects must often wait until a task is completed before deciding whether or not to reallocate those resources. A nurse who is simultaneously managing a number of different patients can only implement the next stage of a treatment protocol for each patient once the previous stage has been completed. An air traffic controller managing competing demands from different aircraft can only perform one task at a time and needs to make choices regarding the order in which those tasks are done. The normative model presented in this article can be extended to continuous tasks, in which people can act on the environment any time (see Lembersky, 1974; Miller, 1968). However, a discrete task is a useful starting point for examining departures from optimality during multiple-goal pursuit, because discrete models provide a useful approximation for continuous processes (Busemeyer & Townsend, 1992; Jagacinski & Flach, 2003), and evidence suggests that the same effects emerge regardless of whether one uses a discrete or continuous task (Brehmer, 1992).

**Figure 1 fig1:**
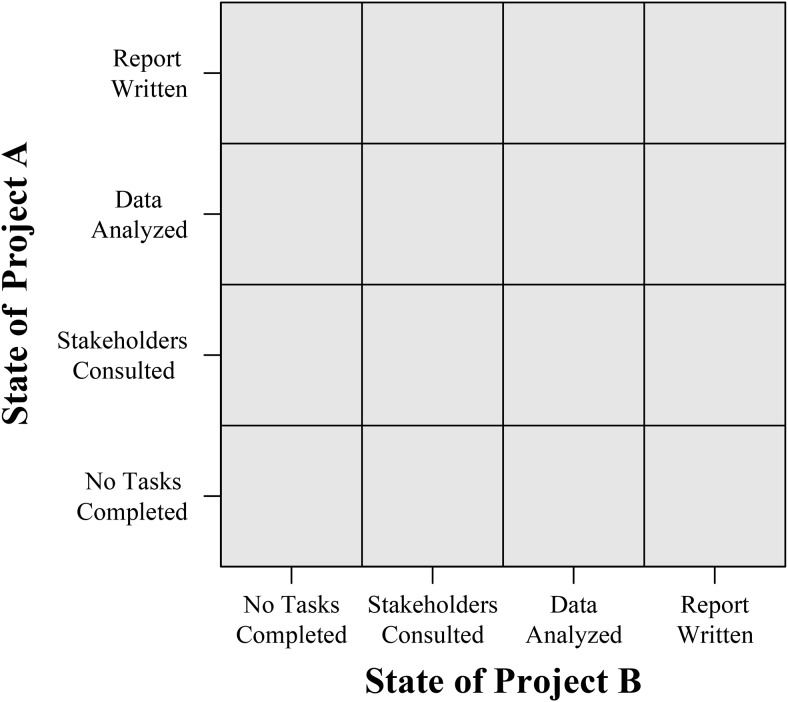
The 16 possible environmental states of a multistage decision task in which a consultant simultaneously strives to complete two projects.

In a multistage decision task, time is conceptualized as discrete steps, which are referred to as *stages*. Consider an example of a consultant who must complete two projects within 10 weeks. Each week, the consultant needs to decide which project gets prioritized. Each week therefore represents a single stage, with the job of completing both projects being broken down into 10 stages in total. When multiple-goal pursuit is conceptualized as a multistage decision task, progress toward each goal determines the *environmental state*. Assume that each project requires a consultant to first consult with stakeholders, then to analyze the data, and finally to write a report. Each project can therefore be in one of four different states. When a project is in its initial state, none of the four tasks will have been completed. In its final state, the report will have been written (and the project therefore completed). Because there are two projects and each can be in any one of the four states, there are 16 possible environmental states (see Figure 1).[Fig-anchor fig1]

## Dynamic Programming

The optimal decision according to expected utility theory (von Neumann & Morgenstern, 1947) is to select the action with the highest expected value. Dynamic programming is a technique used to calculate the expected value of actions in multistage decisions, and thus determine which decisions are optimal (Hutchinson & McNamara, 2000). The optimal decision is determined using backward induction. Broadly, backward induction involves first calculating the optimal decisions at the final stage, because the values of the environmental states that may eventuate after the final decision are known; then using this information to determine the values of environmental states at previous stages, which in turn allows the calculation of the optimal decision at those stages. Dynamic programming is commonly used in the animal behavior literature to determine optimal strategies for carrying out complex sequences of actions (Dall, Houston, & McNamara, 2004; Houston, Clark, McNamara, & Mangel, 1988; McNamara & Houston, 1986). Dynamic programming is also commonly used by decision analysts to find optimal solutions to complex problems in the workplace, such as how to minimize financial losses after an earthquake (Yeo & Cornell, 2009), or how to most efficiently collect meteorological data (Hanlon, Stefik, Small, Verlinde, & Young, 2013).

**Figure 2 fig2:**
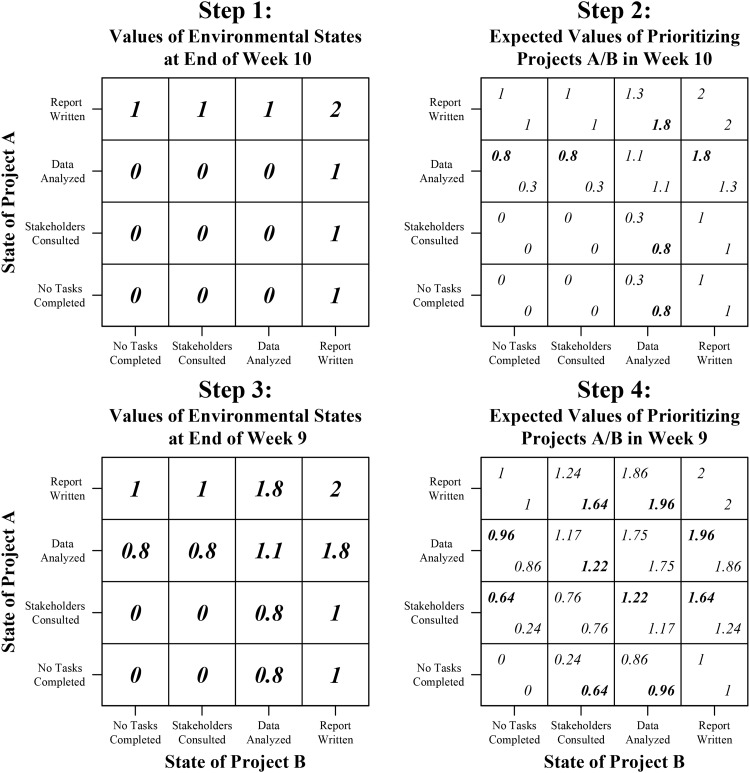
Dynamic programming applied to a multistage decision task in which a consultant simultaneously strives to complete two projects within 10 weeks. The probability of completing a task in a given week on the project that is prioritized is 0.8, and the probability of completing a task on the project that is not prioritized is 0.3. In the diagrams representing Steps 2 and 4, the expected value for prioritizing Project A is shown in the top-left corner of each square; the expected value of prioritizing Project B is shown in the bottom-right corner; and the optimal decision is in bold.

**Figure 3 fig3:**
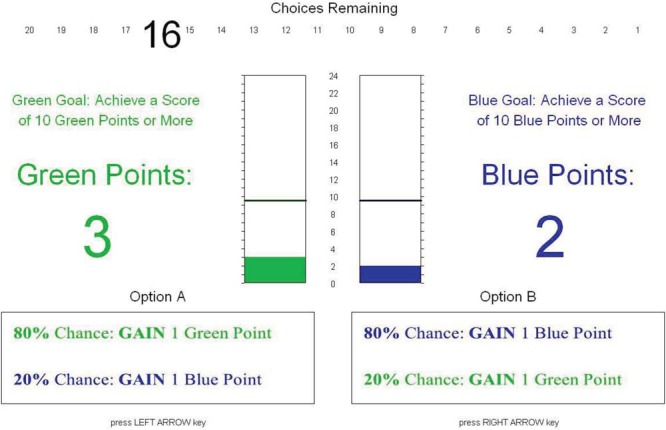
Screen shot of the experimental task (approach condition). See the online article for the color version of this figure.

Figure 2 shows how dynamic programming can be applied to the example described earlier, in which a consultant has to complete two projects within 10 weeks (see Busemeyer et al., 2000; Johnson & Busemeyer, 2001, for further examples). Step 1 is to start after the final week (i.e., Week 10) and identify the value of every environmental state at this point in time. Step 1 in Figure 2 shows the values of each of the 16 states at the end of Week 10. We equate value with the number of attained goals (i.e., the number of projects completed). The state that represents completing both projects therefore has a value of two, the six states that represent completing one project have a value of one, and the nine states that represent neither project being completed have a value of zero.[Fig-anchor fig2][Fig-anchor fig3]

Step 2 involves moving backward to the beginning of Week 10. The task in this step is to identify, for each possible environmental state, whether it is optimal to prioritize Project A or Project B in Week 10. This task is achieved by calculating the expected values (*e*) associated with prioritizing Projects A and B in Week 10 for each environmental state. Here, the expected value of prioritizing a project represents the number of projects that one can expect to complete by the end of Week 10 if that project is prioritized. The optimal decision is to prioritize the project with the highest expected value because this maximizes the number of projects once expects to complete. The expected value of prioritizing each project (*e*_*a*_) is calculated using an expected utility equation:

$$e_{a}(t)=\sum_{s}p_{s|a}v_{s}(t),$$ 1

where *p*_*s|a*_ is the probability of the environmental state resulting from an action being selected (i.e., a project being prioritized), and *v*_*s*_ *(t)* is the value of that environmental state at stage *t* (in this case, stage *t* is Week 10). Step 2 in Figure 2 shows the expected values for prioritizing Projects A or B in Week 10 for each of the 16 possible states. In this example, we have assumed that the probability of a consultant completing a task in a given week on the project that is prioritized is 0.8. There is also a chance that the consultant will make progress on the project that is not prioritized in a given week. For example, while working on one project, the consultant may have an insight for the other project. Alternatively, they may assign a colleague to work on the other project. We assume that the probability of completing a task on the project that is not prioritized is 0.3 (and that whether or not one makes progress toward a goal is independent of progress toward the other goal).

The top-left corner of each square shows the expected value of prioritizing Project A and the bottom-right corner shows the expected value of prioritizing Project B. The optimal decision is in bold. Consider a consultant who, at the beginning of Week 10, only has to write the report for Project A, but has not yet done the data analysis for Project B. The optimal decision for the consultant is to prioritize Project A (expected value = 0.8), because doing so gives a higher chance of completing this project (and thus achieving one of the goals by the end of Week 10) than if he or she were to prioritize Project B (expected value = 0.3).

At Step 3, we start repeating the cycle established in Steps 1 and 2. Step 3 involves identifying the value of each possible environmental state at the end of Week 9. When there is at least one stage remaining, the value of an environmental state is equal to the expected value of the optimal action in the following stage (i.e., the action with the highest expected value). Thus, the value of each environmental state at the end of Week 9 is equal to the expected value of the project that is optimal to prioritize in Week 10. The value of an environmental state when there is at least one week remaining can therefore be represented as follows:

$$v_{s}(t)=\max_{a}[e_{s|a}(t+1)],$$ 2

Step 4 involves moving backward to the beginning of Week 9. Like Step 2, this step involves calculating the expected value of prioritizing each project in Week 9 for every possible state (and therefore the optimal decisions) in this stage using Equation 1. Step 4 in Figure 2 shows these expected values (and optimal decisions in bold). The process outlined in Figure 2 continues backward using repeated cycles of Steps 3 and 4 until the optimal decisions have been determined for every week.

## Approach and Avoidance Goals

In this section, we use prospect theory (Kahneman & Tversky, 1979) to predict departures from optimality when people pursue approach and avoidance goals. According to prospect theory, framing decisions as an opportunity to gain produces risk-averse behavior, whereas framing decisions as a threat of loss produces risk-seeking behavior. For example, people tend to have a risk-averse preference for a disease prevention program in which 200 people are certain to be saved in favor of a program that has a 1/3 chance of saving 600 people (and a 2/3 chance of saving nobody). However, people tend to have a risk-seeking preference for a program that has a 1/3 chance that nobody will die (and a 2/3 that 600 people will die) in favor of a program in which 400 people are certain to die (Tversky & Kahneman, 1981). Given that the different frames contain the same information about probabilities and values, these reversals of preference as a function of framing represent departures from the normative standard.

These arguments suggest that people may depart from optimality in predictable ways during multiple-goal pursuit. When pursuing multiple approach goals (e.g., when given opportunities to gain), prospect theory suggests that individuals should behave in a risk-averse manner. This equates to a bias for prioritizing the goal in the best position, because doing so maintains a chance of attaining one goal while minimizing the risk of failing both goals. This bias should produce a tendency to be less likely to prioritize the goal in the worst position than is optimal. When pursuing multiple avoidance goals (e.g., when faced with threats of loss), individuals should behave in a risk-seeking manner. This equates to a bias for prioritizing the goal in the worst position, because doing so provides the best chance of attaining both goals despite the increased risk of failing both. This bias should produce a tendency to be more likely to prioritize the goal in the worst position than is optimal. The preceding arguments lead to the following prediction.

*Hypothesis:* When pursuing two approach goals, an individual is less likely to prioritize the goal in the worst position than is optimal (i.e., will demonstrate risk-averse behavior); whereas when pursuing avoidance goals, the individual is more likely to prioritize the goal in the worst position than is optimal (i.e., will demonstrate risk-seeking behavior).

## Method

### Participants

The sample consisted of 20 participants (13 males, seven females) with ages ranging from 19 to 61 years (*M* = 27.42, *SD* = 10.09). These individuals were recruited from a mailing list at the University of Bristol and received £10 as compensation, as well as a small performance incentive (explained below).

### Experimental Task

**Figure 4 fig4:**
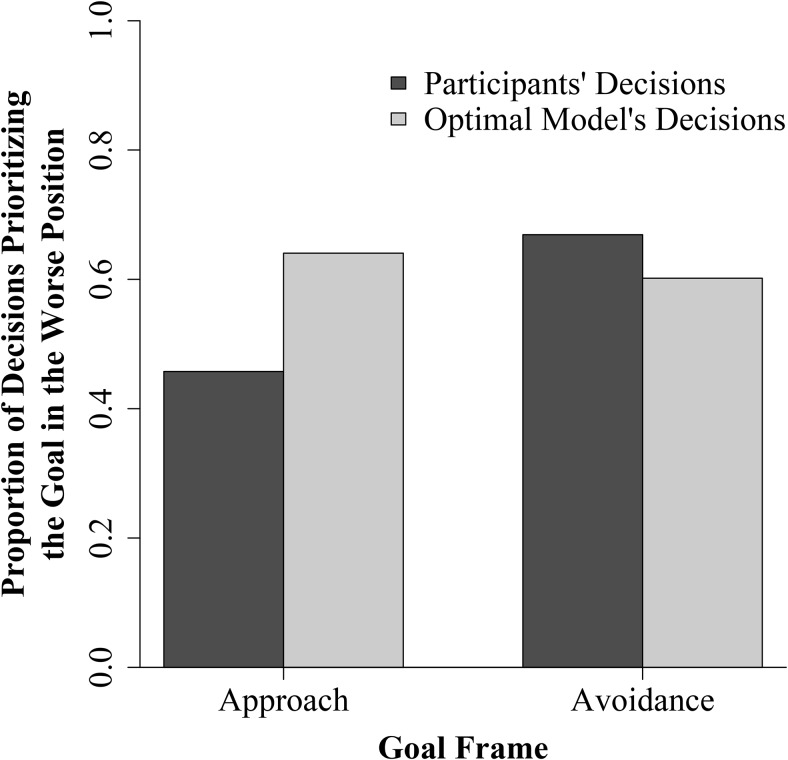
Interaction of goal frame (approach vs. avoidance) and decision source (participants vs. optimal model) on prioritization.

We developed a task that required participants to make a series of discrete prioritization decisions while pursuing two goals (see Figure 3). The task was broken down into a series of trials. Each trial represented a single multiple-goal pursuit episode. Each trial was broken down into a series of decisions (i.e., stages) in which the participant chose between two actions: Option A and Option B. Each action prioritized one goal at the expense of the other, offering an 80% chance of success in making progress (i.e., moving toward an approach goal/not moving toward an avoidance goal) on the prioritized goal and, independently, a 20% chance of success in making progress on the nonprioritized goal. We made participants aware of these probabilities to ensure that departures from optimality were actually due to risk-averse or risk-seeking tendencies, rather than other biases that are known to influence decisions when people are unaware of event probabilities (Camilleri & Newell, 2011). Participants selected an option by pressing the left or right arrow key. They then received immediate feedback about the decision outcome. Participants were then prompted to press the space bar, after which the scores would update and they would be prompted to make the next decision. Participants’ progress through the series of decisions was displayed on the screen and the trial ceased after a prespecified number of actions had been carried out.[Fig-anchor fig4]

### Manipulations

We manipulated goal frame, dual-goal difficulty, and relative position using a (2 × 3 × 4) within-subjects factorial design, producing a total of 24 unique experimental conditions. Each of these manipulations is explained below (see the Appendix for details of each unique condition).

#### Goal frame

We manipulated goal frame within-participants across two levels: approach and avoidance. In the approach condition, the participant had two approach goals: to (a) achieve a score of 10 blue points or more and (b) achieve a score of 10 green points or more. The goals were the same for all trials in the approach condition. Participants began the trial with fewer points than required and had to gain points. In the example shown in Figure 3, Option A prioritizes the blue goal and Option B prioritizes the green goal. If the action was successful with respect to a particular goal, the participant would gain a point for that goal; if unsuccessful, the participant would not gain a point. The outcomes of actions with respect to each goal were independent of each other: Any action could result in gaining a point for both goals, gaining a point for only one goal, or not gaining a point for either goal.

In the avoidance condition, the participants’ two avoidance goals were to (a) avoid a score of 9 blue points or less and (b) avoid a score of 9 green points or less. Participants began the trial with more points than required and had to minimize the loss of points. If the action was successful with respect to a particular goal, the participant would not lose a point for the goal; if unsuccessful, the participant would lose a point.

In addition to their compensation for participating in the study, all participants began the experiment with an extra £4.32 and could gain or lose 3 pence per goal depending on the goal frame condition. The verbal instructions and incentives associated with goal achievement/failure reinforced the goal frame manipulation. In the approach condition, the instructions emphasized the gains associated with goal achievement, and participants could gain money by achieving goals. Before each trial, participants were instructed, “If you achieve both goals, you will GAIN 6 pence. If you achieve one goal, you will GAIN 3 pence. If you do not achieve either goal, you will not gain any money.” After the trial, the participant was informed that “You have gained X pence,” where X was 0, 3, or 6. In the avoidance condition, the instructions emphasized the losses associated with goal failure, and participants could lose money by failing goals. Participants were instructed, “If you fail both goals, you will LOSE 6 pence. If you fail one goal, you will LOSE 3 pence. If you do not fail either goal, you will not lose any money.” After the trial, the participant was informed that “You have lost X pence.”

#### Dual-goal difficulty

We manipulated the difficulty of achieving both goals to produce variability in the optimal decision. When it is easy to achieve both goals, the optimal decision is to prioritize the goal in the worse position because it maximizes the chance of achieving both goals by ensuring that more progress is made on the goal that is further from achievement, while still allowing some progress to be made on the goal that is closer to achievement. When it is difficult to achieve both goals, the optimal decision is to prioritize the goal in the better position because it maximizes the chance of achieving at least one goal by ensuring that most of the progress is made on the goal that is closer to achievement. We manipulated dual-goal difficulty by varying the probability of achieving both goals at the beginning of each trial within participants across three levels (low vs. moderate vs. high). We produced these three levels by varying the starting score and number of decisions a participant could make in each trial. The probability of achieving both goals was between .95 and .96 in the low difficulty condition, .57 and .58 in the moderate difficulty condition, and .03 and .06 in the high difficulty condition.

#### Relative position

We also produced variability in the optimal decision by manipulating the relative position (i.e., the score difference between the two goals) at the beginning of each trial. As the score difference increases, the optimal decision often changes from prioritizing the goal in the worse position to prioritizing the goal in the better position. We manipulated the relative position by varying the score difference at the beginning of each trial within participants across four levels (0, 1, 3, and 5).

### Measures

#### Participant’s decision

Each decision was coded according to whether the participant chose the option that prioritized the goal in the better or worse position, where 1 = worse position and 0 = better position. The dependent variable was coded in this manner because prioritization of the goal in the worst position is a benchmark phenomenon in the literature (e.g., Schmidt & DeShon, 2007).

#### Optimal decision

The decision generated by the optimal model (referred to as the “optimal decision”) was coded in the same way as participants’ decisions (1 = prioritizing the goal in the worse position was optimal, 0 = prioritizing the goal in the better position was optimal). These decisions were calculated using *MATLAB*’s Markov Decision Process toolbox (Chadès, Chapron, Cros, Garcia, & Sabbadin, 2014) with the dynamic programming equations as described in the introduction.

### Procedure

Participants performed the task on computers in an experimental laboratory with an experimenter present at all times. After being presented with instructions, participants completed two practice trials (one approach and one avoidance, both with medium dual-goal difficulty and a starting score difference of 0). Participants then completed all 24 experimental conditions (2 Goal Frame × 3 Dual-goal Difficulty × 4 Relative Position) six times, with a 5-min break halfway through the experiment. Each participant therefore completed 144 trials. The number of decisions in each trial ranged from 11 to 24. In total, each participant made 2,556 decisions.

Participants were presented with information about the goals and monetary incentives prior to each trial. The participants’ goals, scores, number of decisions remaining, and the probabilities that each action would gain or lose points were displayed on screen for the whole trial. After each trial, the participants were presented with feedback reminding them of whether each goal was achieved or failed and informing them of their monetary gain or loss for that trial. After the experiment was completed, the participant received his or her compensation and monetary incentive. The experiment took approximately 90 min to complete. The total number of decisions made across all participants was 51,120 (2,556 decisions per participant × 20 participants). However, because of technical difficulties, data from the first experimental trial were not recorded for two participants. The total number of decisions for which we had data was 51,081.

## Results

We analyzed decisions where participants were faced with a choice between prioritizing the goal in the better position or the worse position. We therefore excluded decisions in which (a) at least one goal had already been either achieved/failed or (b) the scores for each goal were equal. Of the remaining decisions, there were also a small number of cases (i.e., <1%) in which there was no optimal decision because the expected values of the two actions were equal. These cases were also excluded from analysis. The total number of decisions that remained was 28,165, which corresponded to approximately 55% of decisions from any given trial. For each participant decision, we also analyzed the corresponding optimal decision. Thus, the total number of decisions analyzed was 56,330.

We hypothesized that participants would be less likely to prioritize the goal in the worse position than the optimal model when pursuing approach goals but more likely than the optimal model when pursuing avoidance goals. The results are shown in Figure 4. This figure shows the proportion of decisions prioritizing the goal in the worse position as a function of goal frame (approach or avoidance) and decision source (participant or model). We tested for the hypothesized interaction with a logistic mixed-effects model. This model was implemented using the *lme4* package in R (Bates, Maechler, Bolker, & Walker, 2014), by specifying a generalized linear fixed effects model with a logit link function. The dependent variable in the model was whether or not the decision prioritized the goal in the worse position (1 if yes, 0 if no). The predictors were goal frame (1 = approach, −1 = avoidance), decision source (1 = participant, −1 = optimal model), and the interaction between these two variables. The model accounted for the nested structure of the data by controlling for the random effects of participant and trial. The analysis revealed significant main effects of goal frame and decision source, as well as a significant interaction between goal frame and decision source (see Table 1). As Figure 4 illustrates, the interaction was in the predicted direction. People were less likely to prioritize the goal in the worse position than the optimal model when pursuing approach goals but more likely to prioritize the goal in the worse position than the optimal model when pursuing avoidance goals.[Table-anchor tbl1]

We conducted follow-up tests to assess whether the departures from optimality were significant in each condition. To do this, we ran separate logistic mixed-effects models for the approach and avoidance conditions. Each model had a single predictor—decision source. As expected, these analyses revealed that in the approach condition, participants were significantly less likely to prioritize the goal in the worse position than was optimal, β = −0.46, *SE* = 0.01, *p* < .001. In the avoidance condition, participants were significantly more likely to prioritize the goal in the worse position than was optimal, β = 0.09, *SE* = 0.01, *p* < .001. Thus, consistent with our hypothesis, when individuals were pursuing two approach goals, their prioritization decisions were biased in a risk-averse manner, whereas when they were pursuing two avoidance goals, their decisions were biased in a risk-seeking manner.^1^

## Discussion

A growing body of research has focused on identifying factors that predict how people prioritize competing goals (Kernan & Lord, 1990; Louro et al., 2007; Schmidt & DeShon, 2007; Schmidt et al., 2009; Schmidt & Dolis, 2009). However, previous work has not examined whether people make prioritization decisions that are optimal. The optimal choice at any point in time is the one that maximizes the potential for goal achievement. Understanding how people depart from optimality is important because it provides insight into how prioritization decisions are made and facilitates practical efforts to improve them (Baron, 2004, 2012). For this reason, we developed a normative model of prioritization based on expected utility theory and used this model to examine the impact of one factor—goal frame—on the optimality of decisions. In the following sections, we discuss the contributions this work makes to the multiple-goal pursuit literature and suggest avenues for future research. We then discuss the practical implications and potential limitations of this work.

### Departures From Optimality During Multiple Goal Pursuit

One of the primary contributions of the current study is the finding that people depart from optimality in predictable ways during multiple goal pursuit. When pursuing approach goals, people depart from optimality by underprioritizing the goal in the worse position relative to a normative baseline. When pursuing avoidance goals, people depart from optimality in a different way—they overprioritize the goal in the worse position relative to a normative baseline. Previous research in the field has revealed variability in the tendency to prioritize the goal in the worse position when pursuing approach goals (Kernan & Lord, 1990; Schmidt & DeShon, 2007). Schmidt and colleagues have found that this tendency changes as a function of dual-goal difficulty (Schmidt & Dolis, 2009) and the volatility of the environment (Schmidt et al., 2009). We extend this previous work by demonstrating that prioritization depends on the goal frame itself; in our experiment, people prioritized the goal in the worse position about 45% of the time when pursuing approach goals, and about 65% of the time when pursuing avoidance goals. We also extend previous work by demonstrating that these tendencies reflect biases, because they represent systematic departures from optimality. Specifically, people exhibited a risk-averse bias when pursuing approach goals, and a risk-seeking bias when pursuing avoidance goals.

Our findings highlight the value of using theories of decision making to understand multiple-goal pursuit. The risk-averse and risk-seeking biases under approach and avoidance, respectively, are consistent with prospect theory (Kahneman & Tversky, 1979). The support for prospect theory in a dynamic environment is significant because the theory has primarily been applied to static tasks in which the outcomes of decisions do not accumulate over time and individuals are not working toward any specific goal. Only a small number of studies have tested the predictions of prospect theory using dynamic tasks. These studies have revealed that the biases can disappear or even reverse in dynamic environments. For example, Thaler and Johnson (1990) found that in a two-stage gambling task in which participants had no specific goal, people were risk-seeking when making decisions involving gains if they had just experienced a gain (“the house money effect”). By contrast, people were risk-averse when making decisions involving losses if they had just experienced a loss. Hollenbeck et al. (1994) examined risk in a dynamic work simulation task and showed that the effect of framing depends on goal specificity. When given the nonspecific goal to do their best, people made riskier decisions when incentivized with gains as opposed to losses. However, when given a specific goal, consistent with our own findings, people made riskier decisions when incentivized with losses as opposed to gains. These findings, together with our own results, suggest that prospect theory may generalize to dynamic environments in which people are pursuing specific goals.

An important next step is to shift from description to explanation. Our study is descriptive in the sense that it focuses on the factors that produce departures from optimal prioritization. Explanatory models are needed to describe the underlying psychological processes that generate these biases. The multiple-goal pursuit model (Vancouver, Weinhardt, & Schmidt, 2010; Vancouver, Weinhardt, & Vigo, 2014) is currently the only formal model that explains how people make prioritization decisions during multiple goal pursuit. There are a number of ways in which the effects of goal framing might be explained by the multiple-goal pursuit model. Vancouver et al. (2010) proposed that people might perceive avoidance goals as more important than approach goals. If so, then people should be more sensitive to the goal-performance discrepancy for an avoidance goal than an approach goal. This difference in sensitivity may explain why people are more likely to prioritize the goal in the worse position when pursuing avoidance goals than when pursuing approach goals.

Another way to explain the biases observed in the current study might be to incorporate the law of diminishing returns within the multiple-goal pursuit model, in the same way that the law of diminishing returns was incorporated within prospect theory. The law of diminishing returns suggests that the marginal utility of goal achievement should decline with each additional goal achieved in the approach condition. Likewise, the marginal utility of goal failure should decline with each additional goal failed in the avoidance condition. These declines have different implications when pursuing approach and avoidance goals. In the approach context, the pleasure experienced from achieving the first goal is greater than the pleasure experienced from achieving the second goal. Thus, when pursuing approach goals, achievement of the first goal may be perceived as more important, making people less likely to prioritize the goal in the worse position. In the avoidance context, the pain experienced from failing the first goal is greater than the pain experienced from failing the second goal. Thus, when pursuing avoidance goals, avoiding failure of the first goal may be perceived as most important, making people more likely to prioritize the goal in the worse position.

Another potential explanation is dual-goal expectancy. People are more likely to prioritize the goal in the worse position when they believe that they can achieve both goals (Schmidt & Dolis, 2009). It is therefore possible that, in the approach context, the tendency to underprioritize the goal in the worse position was due to people underestimating the likelihood of achieving both goals. Likewise, in the avoidance context, the tendency to overprioritize the goal in the worse position may have been due to people overestimating the likelihood of achieving both goals.

A final potential explanation is time perception. The perception of time available influences prioritization: People prioritize the goal in the worse position less often as they get closer to a deadline (Schmidt & DeShon, 2007). The perception of more time available should therefore increase the likelihood of prioritizing the goal in the worse position. There is some evidence to suggest that people may underestimate the passage of time in avoidance contexts, but overestimate time elapsed in approach contexts (Angrilli, Cherubini, Pavese, & Manfredini, 1997). This disparity in the perception of time may result from people engaging in a more resource-intensive type of information processing under avoidance compared to approach (Roskes, Elliot, Nijstad, & De Dreu, 2013), which can in turn reduce the perception of passing time (Block, Hancock, & Zakay, 2010).

### The Usefulness of Normative Models

This normative model of prioritization is useful for understanding multiple-goal pursuit because it can be used to evaluate optimality in a range of different settings. We have shown that the model can be implemented for multistage decision tasks, in which goal pursuit was broken down into discrete stages and people made a single prioritization decision in each stage. As described in the introduction, multiple-goal pursuit can often be modeled as a multistage decision task. The normative model can also be extended to tasks in which people can reprioritize continuously over time. Identifying the optimal decisions in this type of environment requires a continuous model that accounts for the fact that the environment (and therefore the optimal course of action) can change at any given point in time (see Lembersky, 1974; Miller, 1968). Previous research has found little difference in behavior on dynamic tasks as a function of whether a task is discrete or continuous (Brehmer, 1992). However, a continuous task may enable the examination of other factors that produce departures from optimality. For example, in the absence of discrete decision points, people may vary in how often they switch priority, which may affect the optimality of decision making. For example, an operator who switches from monitoring one system to another too frequently may incur unnecessarily high switch costs, because time will be needed to get up to speed with each system. By contrast, an operator who does not switch frequently enough may fail to notice developing problems in other systems.

Normative models are useful for understanding decision making because they enable the examination of biases, which are systematic departures from the normative model (Baron, 2004; Chapman & Weber, 2006). Biases provide insights into the way that people make decisions (Baron, 2012; Dobbins & Han, 2007). The normative model presented in this article provides the opportunity for new lines of research aimed at understanding other biases that may affect how people make decisions during multiple-goal pursuit. Other biases that might affect multiple-goal pursuit include the sunk-cost effect (Arkes & Blumer, 1985), the status quo bias (Anderson, 2003), and anchoring and adjustment (the overreliance on certain pieces of information; Tversky & Kahneman, 1974). For example, the sunk cost effect refers to the tendency to persist at a task in which one has already invested time or effort (Arkes & Blumer, 1985). This tendency may bias people toward prioritizing goals in which they have invested effort even when the potential for goal achievement is low.

It is an open question whether the biases demonstrated here persist under different conditions. For example, studies of single-stage decisions have revealed mixed evidence about whether decisions differ when outcome probabilities are given, as opposed to when they must be learned—this difference is referred to as the decision-experience gap (Camilleri & Newell, 2011; Hertwig, Barron, Weber, & Erev, 2004). The decision-experience gap exists at least in part because, when decisions are made based on experience, rare events have less impact than they should according to their objective probabilities (Hertwig & Erev, 2009). In the current experiment, participants were informed of the probabilities of various outcomes. One question for future research is whether people make different prioritization decisions when they must learn the probabilities of actions having a particular effect on goal progress. To determine the generalizability of our findings, future research should examine whether people exhibit similar biases in contexts where they must learn the effects of actions (see Satia & Lave, 1973, for generalization of the normative model to this type of environment).

### Practical Implications

There are many situations in which departures from optimality during multiple goal pursuit can have significant consequences. For example, individuals and teams working across multiple projects need to prioritize the allocation of time and effort to meet competing deadlines. People performing process control or scheduling tasks need to prioritize the allocation of physical resources to different components of the system. Managers need to prioritize the allocation of tangible and intangible assets (e.g., capital, finance, human resources) across different business units or ventures. In each case, the suboptimal allocation of resources increases the risk that the decision maker will not achieve one or more of their goals by the deadline.

Understanding how people depart from optimality during multiple-goal pursuit is practically useful because it can be used to inform the development of interventions to improve the quality of decision making during multiple goal pursuit. There are a range of interventions in which dynamic programming can be used, including training (e.g., McGinnis & Fernandez-Gaucherand, 1994), decision support (e.g., Hanlon et al., 2013; Parasuraman, Masalonis, & Hancock, 2000; Yeo & Cornell, 2009), and performance management (e.g., Anderson, 2001; Pinker & Larson, 2003). For example, dynamic programming models can be incorporated into training simulations, and used to provide trainees with feedback that will help them prioritize optimally. Such models can also facilitate the development of decision support systems that improve decision making on the job. Managers may influence the way that staff prioritize the allocation of resources to competing goals by framing those goals in approach or avoidance terms to induce risk aversion or risk-seeking. If the goals cannot be reframed, managers may be able to use instructions or incentives to change the relative importance of the goals, or adjust the difficulty of the goals or the deadlines, to counteract these biases.

### Additional Considerations

To operationalize decision optimality, it is necessary to select a criterion by which to evaluate optimality. We used the criterion of expected-value maximization because it provides an objective, mathematical tool to determine the optimal decision. However, this criterion is not the only way to define optimality. For example, the principle of bounded rationality states that a normative model should take into account the information processing capabilities of the decision maker (Simon, 1955). According to this perspective, expected-value maximization may not be a good criterion for optimality, because processing constraints reduce the amount of information that can be incorporated into the decision. However, it is difficult to use the principle of bounded rationality to derive a normative standard for optimal decision making, because the analyst needs to be able to quantify the amount of information to be processed and the capacity of the human information processor. Despite over 60 years of research on human information processing capacity, this problem has still not been solved (Gopher & Donchin, 1986; Neal et al., 2014)

Another feature of our research that should be considered is the use of a laboratory task and student sample. It is often assumed that student samples lack representativeness, and the results of laboratory experiments lack generalizability. However, such criticisms confuse statistical generalizability with theoretical generalizability. The aim of this research is theoretical generalization—to test a set of hypotheses derived from a theory. A theory can be tested in any sample to which it applies (Highhouse, 2009). Nevertheless, replicating the current study with different tasks, settings, and samples will be necessary to identify potential boundary conditions.

## Conclusion

This research demonstrates the usefulness of theory and methodology from the decision-making literature for understanding multiple-goal pursuit. We have shown how the normative models that have been so useful for understanding basic decision making can help us understand prioritization in dynamic, multiple-goal environments. The finding that people depart from optimality in a manner consistent with prospect theory suggests that basic decision-making principles can provide insight into the complex process of managing competing goals. Although theories of multiple-goal pursuit have begun to integrate accounts of motivation and decision-making (Vancouver et al., 2010), we believe that further integration will lead to a more sophisticated understanding of prioritization. We hope that the continued integration of these distinct theoretical traditions will provide fruitful avenues for future research and practical efforts to enhance decision making in the workplace.

## Figures and Tables

**Table 1 tbl1:** Effects of Goal Frame and Decision Source on Prioritization

Predictor	β	*SE*	*p*
Intercept	−.38	.04	<.001
Goal Frame	−.12	.01	<.001
Decision Source	−.18	.01	<.001
Goal Frame × Decision Source	−.26	.01	<.001

## Footnotes

1 An anonymous reviewer questioned whether our results could be influenced by the two practice trials not having been sufficient to become acquainted with the task or the avoidance condition having been more difficult to understand than the approach. We therefore reanalyzed the data with the first 48 experimental trials (i.e., the first third) excluded and found that the results have the same substantive interpretation as when these trials are included. This finding suggests that learning did not influence the results, as participants would have been very familiar with both conditions by the 48th trial.

## Summary of Manipulations

**Table:**

	Dual-goal difficulty				Goal in better position		Goal in worse position	
Goal frame	Level	Probability of achieving both goals	Number of decisions	Starting score difference	Starting score	Successful actions required	Starting score	Successful actions required
Approach	High	.95	24	0	0	10	0	10
		.95	23	1	1	9	0	10
		.96	21	3	3	7	0	10
		.96	19	5	5	5	0	10
	Moderate	.57	20	0	0	10	0	10
		.57	19	1	1	9	0	10
		.57	17	3	3	7	0	10
		.58	15	5	5	5	0	10
	Low	.06	16	0	0	10	0	10
		.05	15	1	1	9	0	10
		.04	13	3	3	7	0	10
		.03	11	5	5	5	0	10
Avoidance	High	.95	24	0	24	10	24	10
		.95	23	1	24	9	23	10
		.96	21	3	24	7	21	10
		.96	19	5	24	5	19	10
	Moderate	.57	20	0	20	10	20	10
		.57	19	1	20	9	19	10
		.57	17	3	20	7	17	10
		.58	15	5	20	5	15	10
	Low	.06	16	0	16	10	16	10
		.05	15	1	16	9	15	10
		.04	13	3	16	7	13	10
		.03	11	5	16	5	11	10
